# Supplementary material for: Thermodynamics of structure-forming systems
Source: Nat Commun. 2021 Feb 18;12:1127. doi: 10.1038/s41467-021-21272-7 (PMC7893045; doi:10.1038/s41467-021-21272-7)
Supplement: Supplementary file 1 — Supplementary information [file 41467_2021_21272_MOESM1_ESM.pdf]

Supplementary information for “Thermodynamics of structure-forming systems”

Jan Korbel,[1,2] Simon David Lindner,[1,2] Rudolf Hanel,[1,2] and Stefan Thurner[1,2,3,4,*]

[1] *Section for the Science of Complex Systems, CeMSIIS, Medical University of Vienna, Spitalgasse 23, A-1090, Vienna, Austria*
[2] *Complexity Science Hub Vienna, Josefstädterstrasse 39, A-1080 Vienna, Austria*
[3] *Santa Fe Institute, 1399 Hyde Park Road, Santa Fe, NM 87501, USA*
[4] *IIASA, Schlossplatz 1, A-2361 Laxenburg, Austria*

(Dated: January 20, 2021)

This Supplementary Information to the paper *Thermodynamics of structure-forming systems* contains additional information, mainly on details of analytical and numerical computations. It also contains more examples that we mention in the main text.

Supplementary Methods

Equivalence of the exact calculation of the sample space with the grand-canonical ensemble in the thermodynamic limit

Here we show the equivalence of the presented approach with the grand-canonical ensemble in the thermodynamic limit and the limit of low concentrations. Let us consider a chemical reaction, $2X \rightleftharpoons X_2$, with n particles. Let us denote the number of particles X as n_X and the number of molecules X_2 as n_{X_2}. Without loss of generality, let us consider that free particles have an energy ϵ and molecules have zero energy.

Exact calculation: Let us start with entropy

$$S(\wp_X, \wp_{X_2}) = -\wp_X \log \wp_X - \wp_{X_2}(\log \wp_{X_2} + 1) - \wp_{X_2} \log\left(\frac{2}{c}\right) \tag{1}$$

normalization constraint, $\wp_X + 2\wp_{X_2} = 1$, and the energy constraint, $\epsilon \wp_X = \mathcal{U}$.

From this we obtain

$$\wp_X = \exp(-(\alpha) - \beta\epsilon)\,, \tag{2}$$

$$\wp_{X_2} = \frac{c}{2}\exp(-2(\alpha))\,. \tag{3}$$

The Lagrange multiplier α can be calculated from the normalization constraint

$$\exp(-(\alpha) - \beta\epsilon) + 2 \cdot \frac{c}{2}\exp(-2(\alpha)) = 1\,. \tag{4}$$

We obtain two solutions of the quadratic equation, of which only one has a physical meaning, i.e.,

$$\alpha = \log\left(\frac{2ce^{-1+\beta\epsilon}}{-1+\sqrt{1+4ce^{2\beta\epsilon}}}\right)\,. \tag{5}$$

Helmholtz free-energy can be obtained as

$$F = -\frac{\alpha n}{\beta} - \frac{n(\wp_X + \wp_{X_2})}{\beta} = \frac{n}{\beta}\log\left(\frac{-1+\sqrt{1+4ce^{2\beta\epsilon}}}{2ce^{\beta\epsilon-1}}\right) - \frac{n}{\beta}\frac{e^{-2\beta\epsilon}\left(2ce^{2\beta\epsilon} + \sqrt{4ce^{2\beta\epsilon}+1}\right)}{4c} \tag{6}$$

Grand-canonical ensemble: Let’s now compare the exact result with the usual approach using the grand-canonical ensemble. The partition function of the grand-canonical ensemble can be expressed as

$$\mathbb{Z} = \sum_{n_X, n_{X_2}=0}^{\infty} \frac{1}{n_X!}\exp(-\beta(\epsilon - \mu_X)n_X)\frac{1}{n_{X_2}!}\exp(\beta\mu_{X_2} n_{X_2}) = \exp\left(e^{\beta\mu_{X_2}} + e^{-\beta(\epsilon-\mu_X)}\right)\,, \tag{7}$$

where μ_X and μ_{X_2} are the chemical potentials. From the Gibbs-Duhem relation, we get that $\mu_{X_2} = 2\mu_X$. We denote the chemical potential by μ. The average number of particles can be calculated as

$$\langle n \rangle = \frac{\partial \log \mathbb{Z}}{\beta \partial \mu} = 2e^{2\beta\mu} + e^{\beta(\mu-\epsilon)} . \tag{8}$$

This relation serves as an equation for μ, which has the same form as Eq. (4), and the solution can be found as

$$\mu = \frac{\log\left(\frac{-1+\sqrt{1+8\langle n\rangle e^{2\beta\epsilon}}}{4e^{\beta\epsilon}}\right)}{\beta} . \tag{9}$$

Helmholtz free-energy can be expressed from the grand-potential $\Omega = -\beta \log \mathbb{Z}$ as $\mathcal{F} = \Omega + \mu \langle n \rangle$ By plugging in the chemical potential, we obtain that

$$\mathcal{F} = \frac{\langle n \rangle}{\beta} \log\left(\frac{-1\sqrt{1+8\langle n\rangle e^{2\beta\epsilon}}}{4e^{\beta\epsilon}}\right) - \frac{e^{-2\beta\epsilon}\left(4\langle n\rangle e^{2\beta\epsilon} + \sqrt{8\langle n\rangle e^{2\beta\epsilon}+1} - 1\right)}{8\beta} . - \langle n \rangle \log \langle n \rangle n \tag{10}$$

For large $\langle n \rangle$, the fluctuations of particles diminish, so only the states with the average number of particles become relevant and we can set $\langle n \rangle = n$. Moreover, the first term becomes dominant, so

$$F(\beta, \epsilon, n) \approx \mu = \frac{\langle n \rangle}{\beta} \log\left(\frac{-1+\sqrt{1+8\langle n\rangle e^{2\beta\epsilon}}}{4e^{\beta\epsilon}}\right) , \tag{11}$$

and we see that the free-energies of both approaches coincide for $c = n/2$.

Derivation of the second law of thermodynamics for non-equilibrium structure-forming systems

The time derivative of entropy can be expressed as

$$\frac{\mathrm{d}\mathcal{S}}{\mathrm{d}t} = -\sum_{ij} \dot{\wp}_i^{(j)} (\log \wp_i^{(j)} - 1) - \sum_{ij} \dot{\wp}_i^{(j)} - \sum_{ij} \dot{\wp}_i^{(j)} \log\left(\frac{j!}{c^{j-1}}\right) . \tag{12}$$

By plugging in the master equation we can further obtain that

$$\begin{aligned}
\dot{\mathcal{S}} &= -\sum_{ijkl} w_{ik}^{jl} \wp_k^{(l)} \log \wp_i^{(j)} - \sum_{ijkl} w_{ik}^{jl} \wp_k^{(l)} \log\left(\frac{j!}{c^{j-1}}\right) \\
&= +\frac{1}{2}\sum_{ijkl} (w_{ki}^{lj}\wp_i^{(j)} - w_{ik}^{jl}\wp_k^{(l)}) \log \frac{\wp_i^{(j)}}{\wp_k^{(l)}} + \frac{1}{2}\sum_{ijkl} (w_{ki}^{lj}\wp_i^{(j)} - w_{ik}^{jl}\wp_k^{(l)}) \log\left(\frac{j!}{l!} c^{l-j}\right) \\
&= \underbrace{\frac{1}{2}\sum_{ijkl} (w_{ki}^{lj}\wp_i^{(j)} - w_{ik}^{jl}\wp_k^{(l)}) \log \frac{w_{ki}^{lj}\wp_i^{(j)}}{w_{ik}^{jl}\wp_k^{(l)}}}_{\dot{\mathcal{S}}_i \geq 0} + \frac{1}{2}\sum_{ijkl} (w_{ki}^{lj}\wp_i^{(j)} - w_{ik}^{jl}\wp_k^{(l)}) \log\left(\frac{j!}{l!} c^{l-j} \frac{w_{ki}^{lj}}{w_{ik}^{jl}}\right) \\
&= \dot{\mathcal{S}}_i + \underbrace{\frac{\beta}{2}\sum_{ijkl} (w_{ki}^{lj}\wp_i^{(j)} - w_{ik}^{jl}\wp_k^{(l)})(\epsilon_i^{(j)} - \epsilon_k^{(l)})}_{\dot{\mathcal{S}}_e = \beta \dot{\mathcal{Q}}} + \frac{\alpha}{2}\sum_{ijkl} (w_{ki}^{lj}\wp_i^{(j)} - w_{ik}^{jl}\wp_k^{(l)})(j-l) .
\end{aligned} \tag{13}$$

Let us note that from the first law of thermodynamics,

$$\dot{\mathcal{U}} = \sum_{ij} \dot{\wp}_i^{(j)} \epsilon_i^{(j)} + \sum_{ij} \wp_i^{(j)} \dot{\epsilon}_i^{(j)} = \dot{\mathcal{Q}} + \dot{\mathcal{W}} , \tag{14}$$

the entropy flow is equal to the heat flow over the temperature. Let us focus on last term, which can be expressed as

$$\frac{1}{2}\sum_{ijkl} (w_{ki}^{lj}\wp_i^{(j)} - w_{ik}^{jl}\wp_k^{(l)})(l-j) = \sum_{ij} \dot{\wp}_i^{(j)} j \equiv 0 \tag{15}$$

is the time derivative of the normalization condition, i.e., the number of particles in the system and therefore it is identical to zero. Therefore, the second law of thermodynamics can be expressed in the form

$$\frac{\mathrm{d}\mathcal{S}}{\mathrm{d}t} = \dot{\mathcal{S}}_i + \beta \dot{\mathcal{Q}}\,. \tag{16}$$

Derivation of the detailed fluctuation theorem for non-equilibrium structure-forming systems

Let us now focus on the derivation of entropy production along a stochastic trajectory. We define entropy along a stochastic trajectory $|bt = (i(\tau), j(\tau))$ in the following form

$$s(\mathbf{x}(\tau)) = -\left(\log \wp_{i(\tau)}^{(j(\tau))}(\tau) - 1 + \log \frac{(j(\tau))!}{c^{j(\tau)-1}}\right) \tag{17}$$

Let us consider that the stochastic trajectory has jumps at times t_z from (i_z^-, j_z^-) to (i_z^+, j_z^+) with transition rate $w_{i_z^+, i_z^-}^{j_z^+, j_z^-}$. By taking the time derivative we obtain

$$\dot{s} = -\frac{1}{\wp_{i(\tau)}^{(j(\tau))}} \partial_\tau \wp_{i(\tau)}^{(j(\tau))} - \sum_z \delta(\tau - t_z) \log \left(\frac{\wp_{i_z^+}^{(j_z^+)}}{\wp_{i_z^-}^{(j_z^-)}}\right) - \sum_z \delta(\tau - t_z) \log \left(\frac{(j_z^+)!}{(j_z^-)!} \frac{c^{j_z^- - 1}}{c^{j_z^+ - 1}}\right). \tag{18}$$

With a little bit of care, we can recognize that the entropy can be decomposed into two terms. First is entropy flow rate

$$\dot{s}_e = \sum_z \delta(\tau - t_z) \log \left(\frac{w_{i_z^- i_z^+}^{j_z^- j_z^+}}{w_{i_z^+ i_z^-}^{j_z^+ j_z^-}} \frac{(j_z^+)!}{(j_z^-)!} \frac{c^{j_z^- - 1}}{c^{j_z^+ - 1}}\right) = \beta \sum_z \delta(\tau - t_z) (\epsilon_{i_z^-}^{(j_z^-)} - \epsilon_{i_z^+}^{(j_z^+)}) \tag{19}$$

and second, entropy production rate along a stochastic trajectory

$$\dot{s}_i = -\frac{1}{\wp_{i(\tau)}^{(j(\tau))}} \partial_\tau \wp_{i(\tau)}^{(j(\tau))} - \sum_z \delta(\tau - t_z) \log \left(\frac{w_{i_z^- i_z^+}^{j_z^- j_z^+} \wp_{i_z^+}^{j_z^+}}{w_{i_z^+ i_z^-}^{j_z^+ j_z^-} \wp_{i_z^-}^{j_z^-}}\right). \tag{20}$$

Thus, we obtain $\dot{s} = \dot{s}_i + \dot{s}_e$. The ensemble second law of thermodynamics can be recovered by multiplying the trajectory second law by $\wp_i^{(j)}$ and summing over i, j.

Let us consider a stochastic trajectory $\mathbf{x}(\tau) = (i(\tau), j(\tau))$, where $\tau \in [0, T]$. Let us consider that jumps happen at times τ_z, $z \in \{1, \dots, N\}$ from (i_{z-1}, j_{z-1}) to (i_z, j_z). Let us also time-dependent protocol $l(\tau)$ that controls the energy spectrum of the system. We define a quantity

$$\wp(\mathbf{x}(\tau)) = \wp_{i_0}^{(j_0)}(0) \left[\prod_{z=1}^N e^{\int_{\tau_{z-1}}^{\tau_z} \mathrm{d}\tau' w_{i_{z-1} i_{z-1}}^{j_{z-1} j_{z-1}}(l(\tau'))} w_{i_z i_{z-1}}^{j_z j_{z-1}}(l(\tau_z))\right] e^{\int_{\tau_N}^T \mathrm{d}\tau' w_{i_N i_N}^{j_N j_N}(l(\tau'))} \tag{21}$$

that corresponds to a "probability" of the trajectory $\mathbf{x}(\tau)$. We can interpret this quantity as the relative number of clusters in the state $\mathbf{x}(\tau)$ over the total number of particles. Indeed, this quantity does not sum up to one. Similarly, consider reverse trajectory $\tilde{\mathbf{x}}(\tau) = (i(T - \tau), j(T - \tau))$ and reverse protocol $\tilde{l}(\tau) = l(T - \tau)$. We consider microreversibility, i.e., that the detailed balance holds also under the external protocol. Then, we define

$$\tilde{\wp}(\tilde{\mathbf{x}}(\tau)) = e^{\int_{T-\tau_1}^T \mathrm{d}\tau' w_{i_0 i_0}^{j_0 j_0}(\tilde{l}(\tau'))} \left[\prod_{z=1}^N w_{i_{z-1} i_z}^{j_{z-1} j_z}(\tilde{l}(T - \tau_z)) e^{\int_{T-\tau_{z+1}}^{T-\tau_z} \mathrm{d}\tau' w_{i_z i_z}^{j_z j_z}(l(\tau'))}\right] \wp_{i_N}^{(j_N)}(T)\,. \tag{22}$$

The log-ratio of both quantities can be expressed as

$$\begin{aligned}
\log \frac{\wp(\mathbf{x}(\tau))}{\tilde{\wp}(\tilde{\mathbf{x}}(\tau))} &= \log \wp_{i_0}^{(j_0)}(0) - \log \wp_{i_N}^{(j_N)}(T) + \sum_{z=1}^N \log \frac{w_{i_z i_{z-1}}^{j_z j_{z-1}}(l(\tau_z))}{w_{i_{z-1} i_z}^{j_{z-1} j_z}(\tilde{l}(T - \tau_z))} \\
&= \log \wp_{i_0}^{(j_0)}(0) + \log \frac{j_0!}{c^{j_0 - 1}} - \log \wp_{i_N}^{(j_N)}(T) - \log \frac{j_N!}{c^{j_N - 1}} \\
&\quad + \sum_{z=1}^N \log \left(\frac{w_{i_z i_{z-1}}^{j_z j_{z-1}}(l(\tau_z))}{w_{i_{z-1} i_z}^{j_{z-1} j_z}(\tilde{l}(T - \tau_z))} \frac{j_{z-1}!}{j_z!} \frac{c^{j_z - 1}}{c^{j_{z-1} - 1}}\right) = \Delta s - \Delta s_e = \Delta s_i
\end{aligned} \tag{23}$$

Let us now consider the master equation for probability $p_i^{(j)} = j\wp_i^{(j)}$ that a particle belongs to the cluster of size j with energy $\epsilon_i^{(j)}$:

$$\dot{p}_i^{(j)} = \sum_{kl} W_{ik}^{jl} p_k^{(l)} \equiv \sum_{kl} \frac{j}{l}\, w_{ik}^{jl} p_k^{(l)} \tag{24}$$

From this, we obtain that $W_{ik}^{jl} = \frac{j}{l}\, w_{ik}^{jl}$. Thus, the probability of observing the trajectory $\mathbf{x}(\tau)$ with the protocol $l(\tau)$ can be expressed as

$$\mathcal{P}(\mathbf{x}(\tau)) = p_{i_0}^{(j_0)}(0) \left[\prod_{z=1}^{N} e^{\int_{\tau_{z-1}}^{\tau_z} \mathrm{d}\tau' W_{i_{z-1}i_{z-1}}^{j_{z-1}j_{z-1}}(l(\tau'))} W_{i_z i_{z-1}}^{j_z j_{z-1}}(l(\tau_z))\right] e^{\int_{\tau_N}^{T} \mathrm{d}\tau' W_{i_N i_N}^{j_N j_N}(l(\tau'))} \tag{25}$$

and similarly the probability of the time-reversed trajectory under the time-reversed protocol can be written as

$$\tilde{\mathcal{P}}(\tilde{\mathbf{x}}(\tau)) = e^{\int_{T-\tau_1}^{T} \mathrm{d}\tau' W_{i_0 i_0}^{j_0 j_0}(\tilde{l}(\tau'))} \left[\prod_{z=1}^{N} W_{i_{z-1}i_z}^{j_{z-1}j_z}(\tilde{l}(T-\tau_z)) e^{\int_{T-\tau_{z+1}}^{T-\tau_z} \mathrm{d}\tau' W_{i_z i_z}^{j_z j_z}(l(\tau'))}\right] p_{i_N}^{(j_N)}(T)\,. \tag{26}$$

Therefore, the log-ratio can be expressed as

$$\begin{aligned}
\log\frac{\mathcal{P}(\mathbf{x}(\tau))}{\tilde{\mathcal{P}}(\tilde{\mathbf{x}}(\tau))} &= \log p_{i_0}^{(j_0)}(0) - \log p_{i_N}^{(j_N)}(T) + \sum_{z=1}^{N} \log\frac{W_{i_z i_{z-1}}^{j_z j_{z-1}}(l(\tau_z))}{W_{i_{z-1}i_z}^{j_{z-1}j_z}(\tilde{l}(T-\tau_z))} \\
&= \log p_{i_0}^{(j_0)}(0) - \log p_{i_N}^{(j_N)}(T) + \sum_{z=1}^{N} \log\left(\frac{j_z}{j_{z-1}}\right)^2 \frac{w_{i_z i_{z-1}}^{j_z j_{z-1}}(l(\tau_z))}{w_{i_{z-1}i_z}^{j_{z-1}j_z}(\tilde{l}(T-\tau_z))} \\
&= \log p_{i_0}^{(j_0)}(0) - \log p_{i_N}^{(j_N)}(T) + 2\log j_0 - 2\log j_N + \sum_{z=1}^{N} \log\frac{w_{i_z i_{z-1}}^{j_z j_{z-1}}(l(\tau_z))}{w_{i_{z-1}i_z}^{j_{z-1}j_z}(\tilde{l}(T-\tau_z))} \\
&= \log\frac{p_{i_0}^{(j_0)}(0)}{j_0} + \log\frac{j_0!}{c^{j_0}} - \log\frac{p_{i_N}^{(j_N)}(T)}{j_N} - \log\frac{j_N!}{c^{j_N}} \\
&\quad + \log\frac{j_0}{j_N} + \sum_{z=1}^{N} \log\left(\frac{w_{i_z i_{z-1}}^{j_z j_{z-1}}(l(\tau_z))}{w_{i_{z-1}i_z}^{j_{z-1}j_z}(\tilde{l}(T-\tau_z))} \frac{j_{z-1}!}{j_z!}\frac{c^{j_z}}{c_{j_{z-1}}}\right) \\
&= \log\frac{\wp(\mathbf{x}(\tau))}{\tilde{\wp}(\tilde{\mathbf{x}}(\tau))} + \log\frac{j_0}{j_N} = \Delta s_i + \log\frac{j_0}{j_N}\,.
\end{aligned} \tag{27}$$

Therefore, we can write down that

$$\log\frac{\mathcal{P}(\mathbf{x}(\tau))}{\tilde{\mathcal{P}}(\tilde{\mathbf{x}}(\tau))} = \Delta s_i + \log\frac{j_0}{j_N} = \Delta\sigma\,. \tag{28}$$

We can express the probability of observing $\Delta\sigma$ as

$$\begin{aligned}
P(\Delta\sigma) &= \int \mathcal{D}[\mathbf{x}(\tau)]\mathcal{P}(\mathbf{x}(\tau))\delta\left(\Delta\sigma - \log\frac{\mathcal{P}(\mathbf{x}(\tau))}{\tilde{P}(\tilde{\mathbf{x}}(\tau))}\right) \\
&= \exp(\Delta\sigma)\int \mathcal{D}[\tilde{\mathbf{x}}(\tau)]\tilde{P}\left(\tilde{\mathbf{x}}(\tau)\right)\delta\left(-\Delta\sigma - \log\frac{\tilde{\mathcal{P}}\left(\tilde{\mathbf{x}}(\tau)\right)}{\tilde{P}(\mathbf{x}(\tau))}\right) \\
&= \exp(\Delta\sigma)\tilde{P}(-\Delta\sigma)\,.
\end{aligned} \tag{29}$$

This gives us the detailed fluctuation theorem for $\Delta\sigma$.

Let us now assume that the initial state is in equilibrium. We rewrite Δs_i as

$$\Delta s_i = \Delta s - \beta q = \beta(w + q - \Delta f) - \beta q + \Delta m = \beta w - \beta\Delta f\,. \tag{30}$$

Let us now express free-energy f of the equilibrium distribution

$$f(\hat{\wp}_i^{(j)}) = \epsilon_i^{(j)} - Ts(\wp_i^{(j)}) = \epsilon_i^{(j)} + T\left[\left(\log\frac{c^{j-1}}{j!} - j\alpha - \beta\epsilon_i^{(j)}\right) - 1 + \log\frac{j!}{c^{j-1}}\right] = -j\frac{\alpha}{\beta} - \frac{1}{\beta}\,. \tag{31}$$

From the ensemble averaging we obtain that

$$\sum_{ij} \hat{\wp}_i^{(j)} f(\hat{\wp}_i^{(j)}) = -\frac{\alpha}{\beta} \sum_{ij} j \wp_i^{(j)} - \frac{1}{\beta} \sum_{ij} \hat{\wp}_i^{(j)} = -\frac{\alpha}{\beta} - \frac{1}{\beta} \sum_{ij} \hat{\wp}_i^{(j)} = -\frac{\alpha}{\beta} - \frac{\mathcal{M}}{\beta} = \mathcal{F} \, . \tag{32}$$

By plugging (31) into (28) we obtain

$$\frac{\mathcal{P}(\mathbf{x}(\tau))}{\tilde{\mathcal{P}}(\tilde{\mathbf{x}}(\tau))} = \frac{j_0}{j_N} \exp\left(\beta w + (j_f \alpha_f - j_0 \alpha_0)\right) \, . \tag{33}$$

By substitution $j \exp(-\alpha j) = p^{(j)}/\mathcal{Z}_j$, we obtain

$$\frac{\mathcal{P}(\mathbf{x}(\tau))}{\tilde{\mathcal{P}}(\tilde{\mathbf{x}}(\tau))} = \frac{p^{(j_0)}}{p^{(j_f)}} \frac{\mathcal{Z}_{j_f}}{\mathcal{Z}_{j_0}} \exp(\beta w) \tag{34}$$

which can be further rewritten as

$$\frac{\mathcal{P}(\mathbf{x}(\tau)|j_0)}{\tilde{\mathcal{P}}(\tilde{\mathbf{x}}(\tau)|j_f)} = \exp(\beta w - \beta(\Phi_{j_f}(l(T)) - \Phi_{j_0}(l(0)) \tag{35}$$

where $\Phi_j = -\frac{1}{\beta} \log \mathcal{Z}_j$. The probability observing work w starting from an equilibrium state with j_0 can be expressed as

$$\begin{aligned} P_0(w|j_0) &= \int \mathcal{D}[\mathbf{x}(\tau)] \mathcal{P}(\mathbf{x}(\tau)|j_0) \delta \left(\beta w - \beta(\Phi_{j_f} - \Phi_{j_0}) - \log \frac{\mathcal{P}(\mathbf{x}(\tau)|j_0)}{\tilde{\mathcal{P}}(\tilde{\mathbf{x}}(\tau)|j_f)} \right) \\ &= \exp(\beta w - \beta(\Phi_{j_f} - \Phi_{j_0})) \int \mathcal{D}[\tilde{\mathbf{x}}_0(\tau)] \mathcal{P}(\tilde{\mathbf{x}}(\tau)|j_f) \delta \left(-\beta w - \beta(\Phi_{j_0} - \Phi_{j_f}) - \log \frac{\tilde{\mathcal{P}}(\tilde{\mathbf{x}}(\tau)|j_f)}{\mathcal{P}(\mathbf{x}(\tau)|j_0)} \right) \\ &= \exp(\beta w - \beta(\Phi_{j_f} - \Phi_{j_0})) \tilde{P}_0(-w|j_f) \end{aligned} \tag{36}$$

which gives us the Crooks' work fluctuation theorem for structure-forming systems.

Derivation of the self-consistency equation for magnetization in the fully connected Ising model

The free-energy for the fully connected Ising model is given by

$$\mathcal{F} = -\frac{\alpha}{\beta} - \frac{\mathcal{M}}{\beta} \, , \tag{37}$$

where α are the same as for the molecule gas in the magnetic field (see the following section on the molecule gas in the presence of the magnetic field), just with the effective field $h_{eff} = (Jm + h)$. The self-consistency equation is obtained from the relation

$$m = -\frac{\partial \mathcal{F}}{\partial h}|_{h=0} \, , \tag{38}$$

which leads to the following equation

$$m = \frac{\sinh(Jm\beta)}{\sqrt{n + \cosh(Jm\beta)^2}} \left(1 + \frac{n}{\left(\cosh(\beta Jm) + \sqrt{\cosh^2(\beta Jm) + n}\right)^2} \right) \, . \tag{39}$$

This equation has to be solved numerically, similarly to the case of the fully connected Ising model without molecule states. The solution is depicted in Fig. 4 in the main text.

Monte Carlo simulation of the fully connected Ising model

We describe now Monte Carlo simulation applied to a system of free particles with two states $\{\uparrow,\downarrow\}$ and the two-particle molecule with one state $\{\|\}$ and a Hamiltonian $H(n_\uparrow, n_\downarrow, n_\|) = -h(n_\uparrow - n_\downarrow)$. The algorithm puts particles into two boxes, one box for free particles and one box for two particle molecules. The approach is similar to how Panagiotopoulos described in [1]. For example consider boxes, one for "Atoms" and one for two particle molecules. Here Box 1 contains the states $\{\uparrow,\downarrow\}$ and Box 2 the states $\{\|\}$. Two kinds of MC-moves are tried. The first kind of move randomly chooses a particle in the $\uparrow$ or $\downarrow$ state and changes it to the other state. The move is then accepted with the probability $\min(1, \exp(-\beta\Delta H))$. The second kind of move is either dividing a state $\{\|\}$ particle in two states $\{\uparrow,\downarrow\}$, or combining two random particles from Box 1 to a state $\{\|\}$ particle. Which one of the two moves is tried is chosen randomly with equal probability. The division move takes a Box 2 particle (two particle molecule) and deletes it and two new Box 1 particle (one-atomic particles) are generated in its stead. The state of the new particle is either $\uparrow$ or $\downarrow$. Which of the two states the particles are created in is chosen randomly with the probability of the current distribution of the two states in Box 1. The move is then accepted with the probability $\min\left(1, \frac{2n_\| n_\uparrow! n_\downarrow!}{\tilde{n}_\uparrow! \tilde{n}_\downarrow!} \exp(-\beta\Delta H)\right)$, where $\tilde{n}_\uparrow$ and $\tilde{n}_\downarrow$ are the numbers for $\uparrow$ and $\downarrow$ particles after the move, respectively. The combination move takes two random particles in Box 1 deletes them and creates a new particle in Box 2. The move is then accepted with $\min\left(1, \frac{n_\uparrow! n_\downarrow!}{\tilde{n}_\uparrow! \tilde{n}_\downarrow! (2n_\| + 2)} \exp(-\beta\Delta H)\right)$. Here, $\tilde{n}_\uparrow$ and $\tilde{n}_\downarrow$ are once again the number of $\uparrow$ and $\downarrow$ particles after the move, respectively. The simulation ends after the ensemble does not change significantly anymore and reaches an equilibrium. The final output is the mean over 1000 simulations at a certain temperature value. Each simulation consists of 2 million steps.

Supplementary discussion

Relation of the entropy for molecule system to axiomatic frameworks

In this section, we discuss the relation of the entropy for molecule systems to existing axiomatic frameworks, including axiomatics of Shannon and Khinchin and its generalizations according to Tempesta and Jensen, and Hanel and Thurner, and also axiomatics according to Shore and Johnson.

Lieb-Yngvason axioms Let's discuss the main properties of the entropic functional (9) from the main text. We start with *additivity* and *extensivity*, as introduced by Lieb and Yngvason [2]. *Additivity* can be formulated as $S((X,Y)) = S(X) + S(Y)$ (see Eq. (2.4) in [2]) where (X,Y) is a cartesian product of two systems, i.e., a state of the composed systems is a pair (x,y), where $x \in X$ and $y \in Y$. We consider two systems, one with χ particles, the other with ξ particles. For simplicity, consider that the particles can attain the same states for both systems. We denote the number of clusters in state $x_i^{(j)}$ in the first and second subsystem as $\chi_i^{(j)}$ and $\xi_i^{(j)}$, respectively. Since the two subsystems are independent, the total multiplicity for $n_i^{(j)} = \chi_i^{(j)} + \xi_i^{(j)}$ is simply given by the product of two multiplicities, so that

$$W(n_i^{(j)}) = W(\chi_i^{(j)}) W(\xi_i^{(j)}) \,, \tag{40}$$

from which we immediately see that $S(n_i^{(j)}) = S(\chi_i^{(j)}) + S(\xi_i^{(j)})$. The second property, *extensivity*, states that $S(tX) = tS(X)$, where tX is the rescaled version of the system (see Eq. (2.5) in [2]). For simplicity, let us consider that we double the system, i.e., $t = 2$. This means that we have $2n$ particles and there are $2n_i^{(j)}$ particles in the state $x_i^{(j)}$. We also have to double the systems' volume, i.e., we divide the total system into $2b$ boxes. The entropy of the double system is then equal to

$$\begin{aligned} S(2n) = 2n \log \frac{2n}{2b} - 2n - \sum_{ij} 2n_i^{(j)} \left(\log \frac{2n_i^{(j)}}{2b} - 1 \right) \\ - \sum_{ij} 2n_i^{(j)} \log j! = 2S(n) \,. \end{aligned} \tag{41}$$

Thus, the entropy is extensive. Another important property is *concavity* of entropy, ensuring the uniqueness of the maximum entropy principle. Since it is straightforward to show that

$$\frac{\partial^2 S(P)}{\partial p_i^{(j)} \partial p_{i'}^{(j')}} = -\frac{1}{j p_i^{(j)}} \delta_{ii'} \delta_{jj'} \,, \tag{42}$$

we conclude that the entropy is a *concave* function of probability distribution.

Classes of entropies and joint entropies: Before we move to particular axiomatic frameworks based on information-theoretic approaches, let us discuss typical classes of entropies that are taken into account. These are

1. *Trace-class entropies* $S(P) = \sum_i g(p_i)$
2. *Sum-class entropies* $S(P) = f(\sum_i g(p_i))$.

These entropies are widely used in information theory and statistical physics because of its nice properties. However, neither of the classes is suitable for our case. One of the issues that occur is that these entropy classes are symmetric functions of all probabilities, which is a consequence of the principle that relabeling the states should not change the entropy (also called permutational invariance - see the next section about Shore-Johnson axioms). It is, however, not the case of a system with molecule states. Here, switching the order of molecules (e.g., from free particle states to molecule states) changes the system's entropy since the states corresponding to molecules of different orders are states of different types, and one cannot expect that the symmetry argument holds.

There is another assumption that is implicitly considered by these classes of entropy. Namely, it is the assumption that the *joint entropy*, i.e., the entropy of the joint distribution has the same functional form as the entropy of the marginal distribution, i.e., in the case of the joint probability of two random variables, we have

$$S(p_{ij}) = f(\sum_{ij} g(p_{ij})) \tag{43}$$

This assumption makes perfect sense for systems with exponential sample spaces, where the joint distribution can also be interpreted as a marginal distribution of a system that is obtained by merging the two systems together, i.e., $X \times X \sim 2X$, where on the left-hand side is the cartesian product of two systems and on the right-hand side is the rescaled version of the system. However, in our case $W(2n) \geq W(n)^2$ so there is no such correspondence.

Let us demonstrate this on an example of two systems A and B with n and m molecules. Corresponding probability distributions are $\tilde{u}_i^{(j)}$ and $\tilde{v}_{i'}^{(j')}$. The entropy of the composed system $(A, B) \equiv A \times B$ is given by the sum of the entropies of two systems, as demonstrated above. If we try to express this entropy in terms of joint distribution $\tilde{p}_{ii'}^{(jj')} = \tilde{u}_i^{(j)} \tilde{v}_{i'}^{(j')}$ we obtain

$$\begin{aligned} S(A,B) &= -\sum_{ij} \frac{\tilde{u}_i^{(j)}}{j} \left(\log \frac{\tilde{u}_i^{(j)}}{j} - 1 \right) - \sum_{ij} \frac{\tilde{u}_i^{(j)}}{j} \log \left(\frac{j!}{c_A^{j-1}} \right) \\ &\quad - \sum_{i'j'} \frac{\tilde{v}_{i'}^{(j')}}{j'} \left(\log \frac{\tilde{v}_{i'}^{(j')}}{j'} - 1 \right) - \sum_{i'j'} \frac{\tilde{v}_{i'}^{(j')}}{j'} \log \left(\frac{j'!}{c_B^{j'-1}} \right) \\ &= -\sum_{iji'j'} \tilde{p}_{ii'}^{(jj')} \left(\frac{1}{j} \log \frac{\sum_{i'j'} \tilde{p}_{ii'}^{(jj')}}{j} - 1 + \frac{1}{j'} \log \frac{\sum_{ij} \tilde{p}_{ii'}^{(jj')}}{j'} - 1 \right) \\ &\quad - \sum_{iji'j'} \tilde{p}_{ii'}^{(jj')} \left(\frac{1}{j} \log \frac{j!}{c_A^{j-1}} + \frac{1}{j'} \log \frac{j'!}{c_B^{j'-1}} \right) \end{aligned} \tag{44}$$

So we see that the joint entropy is expressible in terms of the joint distribution, but the functional form is different from the entropy of the marginal distribution.

Let us note that the composed system created from systems with n_1 particles and n_2 particles is, in general, different from a system with $n_1 + n_2$ particles. Therefore, one cannot expect that the system's entropy can be obtained as a sum of the subsystems. The system's state space with $n_1 + n_2$ particles cannot be represented as a cartesian product of states from the subsystems. A simple example can exemplify this issue: we can consider a molecule state consisting of two particles in a system. The first particle is taken from the first n_1 particles, and the other particle is taken from the remaining n_2 particles. Such a state has no representation in the Cartesian product. It is the consequence of the fact that the sample space grows super-exponentially, and therefore $W(n) > W(n_1)W(n_2)$.

Shannon-Khinchin axioms: Shannon-Khinchin (SK) axioms characterize the properties of the Shannon entropy from the information-theoretic point of view. They were proposed independently by Shannon [3] and Khinchin [4] to determine the Shannon entropy uniquely. In the original formulation, the Shannon-Khinchin axioms are the following:

1. *Continuity:* Entropy is a continuous function of probability distribution.

2. *Maximality:* Entropy is maximal for the uniform distribution.

3. *Expansibility:* Adding an elementary event with probability zero does not change the entropy.

4. *Additivity:* $H(A, B) = H(A) + H(B|A)$, where $H(B|A) = \sum_i p_i H(B|A = a_i)$.

Since the original four axioms uniquely determine Shannon entropy $H(P) = -\sum_i p_i \log p_i$, several authors proposed generalizations of the original scheme. The typical approach is to weaken the fourth axiom to obtain a wider class of entropies while the first three axioms remain unchanged. Thus, before discussing the generalizations of the fourth SK axiom, let us focus on the first three axioms.

It is easy to show that the molecule entropy fulfills the first and the third axiom. As we have discussed in the main text, the entropy is not maximized by the uniform distribution when molecules of different sizes are present. The maximality axiom results from a similar requirement, i.e., that the entropic functional should be symmetric and Schur-concave function of the probability distribution. In the last section, we have discussed that the assumption of symmetry is not suitable for our system. Therefore, it is more natural to weaken the maximality axiom to the following form:

2. *Maximality:* Entropy is maximal for a distribution, where each microstate contributes with equal probability.

By a generalization of SK2, the entropy of molecule states fulfills the first three SK axioms, plus the first part of the fourth one, i.e., $S(A \times B) = S(A) + S(B)$, where $A \times B$ is the Cartesian product of random variables. It means that the state space is a Cartesian product of A and B and joint probability is simply a marginal probabilities product, as demonstrated in the previous section, when we discussed entropy's additivity. Let us now omit discussion about the definition of conditional probability, which is quite technical and would deserve a separate paper.

Tempesta axioms and group entropies: Tempesta proposed in the series of papers [5–7] a generalization of SK axioms, but weakening the fourth axiom by imposing that the entropy should fulfill the group property:

4. *Group composability:* $S(A, B) = \Phi(S(A), S(B))$

where $x, y \mapsto \Phi(x, y)$ is the group action. In the previous section, we have shown that the entropy of molecule systems is additive and therefore fulfills the composability property with $\Phi(x, y) = x + y$. However, the whole calculation was done in the framework of sum-class of entropies. Therefore, the entropy of structure-forming systems does not fall into this reduced class of entropies that work with states of the same structure. However, when relaxing the second SK axiom and omitting the requirement of symmetric entropies, the entropy of molecule systems belongs to the class of group entropies. Relaxing 2nd SK axiom in the framework of group entropies would an important step in the future research.

Hanel-Thurner axioms and entropy scaling: Hanel and Thurner generalized the fourth axiom differently. They did not require any particular composition law; they only examined the asymptotic scaling of the entropy for the case of distribution that maximizes the entropy as a function of the system size (here the number of particles) [8, 9]. The resulting classification leads to the set of scaling exponents (originally (c, d)) that determined universality classes of entropic functionals. Since the entropic functionals are considered to be trace-class, asymptotic scaling was examined for uniform distribution. Nevertheless, for the entropy of structure-forming systems, the distribution that maximizes the entropy does not have to be uniform. Actually, by plugging the MaxEnt distribution into the entropy of molecule systems, we obtain that $S(n) \sim \log n$, so we obtain that $(c, d) = (0, 1)$, which is the case of additive entropies (including Shannon entropy).

Shore-Johnson axioms: Shore and Johnson considered the principle of maximum entropy as a statistical inference method and formulated a set of consistency requirements [10, 11]. They considered the class of inductive inference (i.e., they are in the form of averaged quantities). The requirements are the following:

- *Uniqueness:* the result should be unique.

- *Permutation invariance:* the permutation of states should not matter.

- *Subset independence:* It should not matter whether one treats disjoint subsets of system states in terms of separate conditional distributions or in terms of the full distribution.

- *System independence:* It should not matter whether one accounts for independent constraints related to independent systems separately in terms of marginal distributions or in terms of full system.

- *Maximality:* In absence of any prior information, the uniform distribution should be the solution.

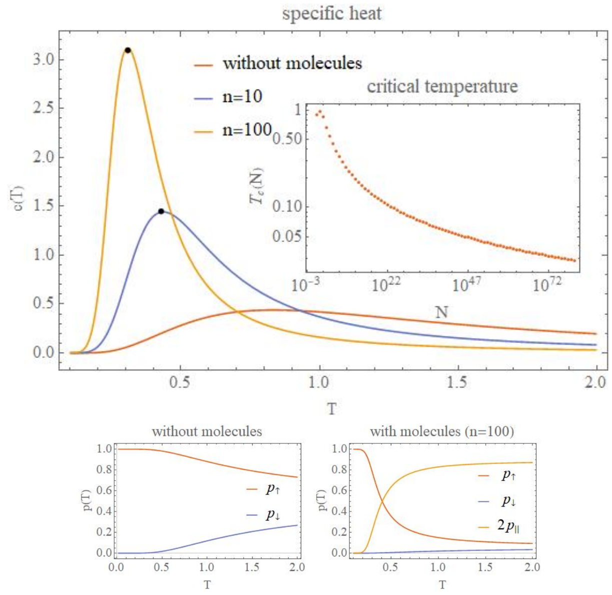

Supplementary Figure 1: Specific heat of the molecule model with magnetic field. We observe a phase transition for the magnetic model. The critical temperature decreases with n to zero very slowly (inset).

In [11] was shown, these axioms are equivalent to Tempesta's group composability under the assumption of sum-class entropies. Indeed, some of the axioms are not fulfilled for the case of molecule entropy. Especially axioms 2 and 5 are other forms of the same assumption that entropy should be a symmetric function of its variables. Therefore, a generalization of SJ axioms for state spaces with states of different types should be reasonable.

Presence of magnetic gas phase for molecule-forming particles in presence of magnetic field

Let us consider a system of n particles where free particles can have two states $\{\uparrow,\downarrow\}$ and two-particle molecules have one state $\{\|\}$. Let us assume the case when the system is small and dense so that all particles can interact with each other. The Hamiltonian corresponding to the magnetic field is

$$H(n_\uparrow, n_\downarrow, n_{\|}) = -h(n_\uparrow - n_\downarrow)\,. \tag{45}$$

We calculate the specific heat $c = -T\frac{\mathrm{d}^2 F}{\mathrm{d}T^2}$ and we see that there is a phase transition between the magnetic phase and molecule phase which grows with n, as shown in Fig. 1 Let us note that the dependence of critical temperature on n, where $\lim_{N\to\infty} T_c(N) = 0$. However, as shown in the inset, the critical temperature is well separated from zero even for large, but finite systems, since the convergence is very slow. It should be mentioned that the magnetic gas has been observed for low temperatures experimentally [12, 13].

Finite-size correction to chemical potential

Let us consider again the chemical reaction $2X \rightleftharpoons X_2$. Without loss of generality, assume that free particles carry some energy ϵ. The equilibrium constant of the chemical reaction can be expressed as

$$K_c = \frac{n_{X_2}}{n_X^2} = \frac{\wp_{X_2}}{(\wp_X)^2} = \exp\left(2\beta\epsilon + \log\frac{c}{2}\right)\,. \tag{46}$$

Thus, we obtain the effective chemical potential, $\Delta\mu^{eff} = 2\epsilon + \frac{1}{\beta}\log\frac{c}{2}$, where the first term can be obtained from the ordinary grand-canonical ensemble of two-gas system and the second one is the correction which is obtained from the molecule entropy. This means that if the two gases are perfectly mixed in a small region so that every particle can interact with each other particle, the value of the chemical potential explicitly depends on the number of particles — with an increasing number of particles the chemical potential increases. In fact, the finite-size corrections to the

chemical potential have been considered in several aspects, especially in case of interacting particles [14, 15]. In our case, the correction is simply because of the structure-forming states.

Supplementary References

* Correspondence to: stefan.thurner@meduniwien.ac.at

[1] A. Z. Panagiotopoulos, Monte Carlo methods for phase equilibria of fluids. *J. Phys.: Condens. Matt.* **12(3)** (1999) 25-52.
[2] E. H. Lieb and J. Yngvason, The physics and mathematics of the second law of thermodynamics. *Phys. Rep.* **310(1)** (1999) 1-96.
[3] C. Shannon, A mathematical theory of communication. *Bell Syst. Tech. J.* **27** (1948) 379.
[4] A.I. Khinchin, Mathematical Foundations of Information Theory. Dover Publications, New York (1957).
[5] P. Tempesta, Beyond the Shannon–Khinchin formulation: The composability axiom and the universal-group entropy. *Ann. Phys.* **365** (2016) 180.
[6] H. J. Jensen and P. Tempesta, Group entropies: From Phase Space Geometry to Entropy Functionals via Group Theory. *Entropy* **20** (2018) 804.
[7] P. Tempesta and H. J. Jensen, Universality Classes and Information-Theoretic Mesure of Complexity via Group Entropies. *Sci. Rep.* **10** (2020) 5952.
[8] R. Hanel and S. Thurner, A comprehensive classification of complex statistical systems and an axiomatic derivation of their entropy and distribution functions. *Europhys. Lett.* **93** (2011) 20006.
[9] J. Korbel, R. Hanel and S. Thurner, Classification of complex systems by their sample-space scaling exponents. *New J. Phys.* **20** (2018) 093007.
[10] P. Jizba and J. Korbel, Maximum Entropy Principle in Statistical Inference: Case for Non-Shannonian Entropies. *Phys. Rev. Lett.* **122** (2019) 120601.
[11] P. Jizba and J. Korbel, When Shannon and Khinchin meet Shore and Johnson: Equivalence of information theory and statistical inference axiomatics. *Phys. Rev. E* **101** (2020) 042126.
[12] G.-B. Jo, Y.-R. Lee, J.-H. Choi, C. A. Christensen, T. H. Kim, J. H. Thywissen, D. E. Pritchard, and W. Ketterle, Itinerant Ferromagnetism in a Fermi Gas of Ultracold Atoms. *Science* **325** (2009) 5947.
[13] R. A. Duine and A. H. MacDonald Itinerant Ferromagnetism in an Ultracold Atom Fermi Gas.- *Phys. Rev. Lett.* **95** (2005) 230403.
[14] B. Smit and D. Frenkel, Explicit expression for finite size corrections to the chemical potential. *J. Phys.: Condens. Matt.* **1** (1989) 8659.
[15] J. I. Siepmann, I. R. McDonald, and D. Frenkel, Finite-size corrections to the chemical potential. *J. Phys.: Condens. Matt.* **4(3)** (1992) 679.
